# Acclimation Training Improves Endurance Cycling Performance in the Heat without Inducing Endotoxemia

**DOI:** 10.3389/fphys.2016.00318

**Published:** 2016-07-29

**Authors:** Joshua H. Guy, David B. Pyne, Glen B. Deakin, Catherine M. Miller, Andrew M. Edwards

**Affiliations:** ^1^Department of Sport and Exercise Science, James Cook UniversityCairns, QLD, Australia; ^2^Faculty of Sport and Health Sciences, University of St Mark & St JohnPlymouth, UK; ^3^Department of Physiology, Australian Institute of SportCanberra, ACT, Australia; ^4^Biomedical Sciences, College of Public Health, Medical and Vet Sciences, James Cook UniversityCairns, QLD, Australia

**Keywords:** cycling, heat acclimation, inflammation, lipopolysacharide, cytokine, endurance performance

## Abstract

**Purpose:** While the intention of endurance athletes undertaking short term heat training protocols is to rapidly gain meaningful physical adaption prior to competition in the heat, it is currently unclear whether or not this process also presents an overt, acute challenge to the immune system. The aim of this study was therefore to examine the effects of heat training on both endurance performance and biomarkers associated with inflammatory and immune system responses.

**Methods:** Moderately-actively males (*n* = 24) were allocated randomly to either HOT (*n* = 8, 35°C, and 70% RH; NEUTRAL (*n* = 8, 20°C, and 45% RH); or a non-exercising control group, (CON, *n* = 8). Over the 18 day study HOT and NEUTRAL performed seven training sessions (40 min cycling at 55 of *V*O_2_ max) and all participants completed three heat stress tests (HST) at 35°C and 70% RH. The HST protocol comprised three × sub-maximal intervals followed by a 5 km time trial on a cycle ergometer. Serum samples were collected before and after each HST and analyzed for interleukin-6, immunoglobulin M and lipopolysaccharide.

**Results:** Both HOT and NEUTRAL groups experienced substantial improvement to 5 km time trial performance (HOT −33 ± 20 s, *p* = 0.02, NEUTRAL −39 ± 18 s, *p* = 0.01) but only HOT were faster (−45 ± 25 s, and −12 s ± 7 s, *p* = 0.01) in HST_3_ compared to baseline and HST_2_. Interleukin-6 was elevated after exercise for all groups however there were no significant changes for immunoglobulin M or lipopolysaccharide.

**Conclusions:** Short-term heat training enhances 5 km cycling time trial performance in moderately-fit subjects by ~6%, similar in magnitude to exercise training in neutral conditions.Three top-up training sessions yielded a further 3% improvement in performance for the HOT group. Furthermore, the heat training did not pose a substantial challenge to the immune system.

## Introduction

Short- and medium-term heat acclimation training protocols are widely used by endurance athletes to increase both heat tolerance and subsequent competitive performances in the heat (Périard et al., 2015). Although favorable performance and physiological benefits can be realized from short term programs (≤ 7 days) (Garrett et al., 2011; Chalmers et al., 2014), greater benefits are likely from longer protocols (7–14 days) (Nielsen et al., 1997; Lorenzo et al., 2010; Daanen et al., 2011; Guy et al., 2015). For elite athletes, busy training, and performance schedules limit the time is available for strategies such as heat training, and addition of supplementary training sessions may sustain and/or complement the initial adaptations.

While the acute effects of short-term heat exposure on blood biomarkers associated with inflammation have been reported (Hailes et al., 2011; Gill et al., 2014), few studies have investigated the effects of longer duration heat training. The human immune system can usually deal with mild-to-moderate inflammatory responses, however, when a heat stimulus is too large, systemic inflammation can result in heat shock and potentially fatal sepsis (Bouchama et al., 2007). Athletes will generally seek a heat training stimulus that is large enough to evoke a training adaptation; however, there likely comes a point where the risk of clinical or subclinical levels of immune disturbance increases.

Exercise-induced endotoxemia is a potential risk of strenuous activity in the heat primarily attributed to translocation of lipopolysaccharide (LPS) from the gut into the circulation (Lim et al., 2009). An abundance of circulating LPS can evoke an inflammatory response, leading to heat shock, and overwhelming anti-LPS mechanisms including immunoglobulin M (IgM) (Camus et al., 1998) and cytokines operating in an anti-inflammatory role such as interleukin-6 (IL-6; Abbasi et al., 2013). Consequently, when anti-LPS mechanisms and rate of LPS clearance are inadequate to counter the heat-induced increase of LPS, endotoxemia may ensue. This outcome could potentially occur during a period of heat acclimation training if the athlete is unable to cope with the thermal loads presented. As IgM is a key antibody in neutralizing LPS (Camus et al., 1998), its concentration in circulating blood can reflect the body's response to endotoxin accumulation, and the degree of protective capacity in the event of further challenges. IgM concentration can increase substantially (~20%) after exercise in the heat, although this elevation does not occur following 5 days of heat training (Hailes et al., 2011). Of the few studies that have investigated IL-6 as a blood biomarker during exhaustive exercise in the heat, Selkirk et al. (2008) observed a 20-fold increase in plasma concentrations following 2 h of exhaustive walking in protective clothing in very hot and humid conditions, with IL-6 inhibiting endotoxin induced increases in tumor necrosis factor alpha and cytokines. Furthermore, the neuroinflammatory response to exercise indicates that an increase in cytokine concentration such as IL-6 reaching a critical threshold, it is likely that sensations of fatigue develop to prevent traumatic injury of specific organs and other physiological systems within the body (Vargas and Marino, 2014). Therefore, athletes who undertake short or medium duration heat acclimation training programs could potentially be at increased risk of exercise-induced heat stress and immune disturbances associated with fatigue.

Recreationally-active healthy adults often participate in one-off events such as an ironman triathlon, marathon and week-long sporting events such as the Masters' Games. It appears that the threshold for the onset of exercise-induced endotoxemia is lower in untrained than trained individuals (Selkirk et al., 2008). Individuals seeking to use heat acclimation training as an additional training stimulus may choose either a short- or medium-term program, to elicit the classic thermal markers of plasma volume expansion, lower heart rate at submaximal intensities and lower end point core temperature, which collectively promote aerobic performance (Guy et al., 2015). However, addition of a heat load to training can often be very demanding, with some studies implementing challenging protocols on their participants, e.g., 90 min of cycling for 10 consecutive days (Gibson et al., 2015). It is prudent to account for both training load and accumulated inflammation from heat stress over the training period. As longer heat training sessions (>60 min) are likely fatiguing for recreationally-trained athletes, and can increase peripheral fatigue compared with shorter protocols (Wingfield et al., 2016), the addition of shorter and supplementary training sessions could yield similar benefits, but with lower overall stress.

Few studies have investigated the degree of inflammation and endotoxemia associated with short- and medium-term heat acclimation training. Therefore, the aim of this study was to investigate whether short-term heat training with the addition of supplementary sessions can improve cycling time trial (TT) performance, improve sub-maximal exercising heart rate and core temperature, and to quantify the degree of inflammation associated with heat acclimation training.

## Methods

### Design

This study consisted of three groups of recreationally-active male athletes: a heat training group (HOT), a matched thermo-neutral training group (NEUTRAL), and a control (no training) group (CON), in a pre–post parallel groups design.

### Participants

Twenty-four moderately trained male participants (3 ± 1 moderate-high intensity training sessions per week, duration 60 ± 15 min; mean ± SD) aged 24.5 ± 3.8 years, height 178 ± 7 cm, mass 84.6 ± 10.8 kg, body fat 17.5 ± 6.1%, and maximal oxygen uptake (*V*O_2_ max) of 45.0 ± 5.0 ml.kg.min^−1^ volunteered for the study. Prior to taking part, participants provided written informed consent in accordance with the Declaration of Helsinki and underwent a pre-screening health questionnaire including use of anti-inflammatory or immunomodulating medications (none were present). The study protocol was approved by the James Cook University Human Research Ethics Council (Approval number H5647).

### Methodology

**Figure 1:**
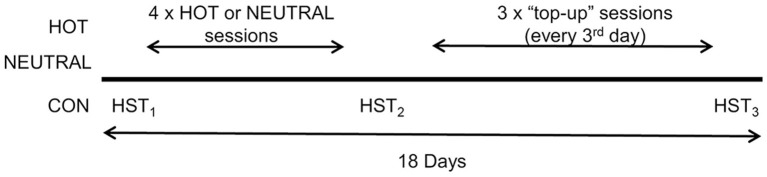
**Study timeline for Heat Training (HOT), Thermo-neutral Training (NEUTRAL), and Control (CON) groups**.

Assessment of *V*O_2_ maxwas undertaken on a cycle ergometer (VeloTron and Velotron Coaching Software, Racermate, United States) at least 72 h before beginning the experimental trials. The intervention comprised a short-term training protocol of four training sessions on consecutive days, followed by three supplementary training sessions every 3 days. All participants completed three heat stress tests (HST_1−3_) and seven training sessions over 18 days, with HST_1_ performed as a baseline measure of heat tolerance, HST_2_ completed between the end of the short-term program and before beginning the supplementary top-up training, and HST_3_ completed 48 h after the final supplementary training session (Figure 1). Each group performed the HST in a custom-built environmental chamber at a temperature of 35°C and 70% RH. Participants in the HOT and NEUTRAL conditions completed exercise training sessions in hot and humid (35°C and 70% RH) or thermo-neutral conditions (20°C and 50% RH), respectively. Participants in the CON group did not undertake exercise training but completed the three HST's at the same intervals as HOT and NEUTRAL groups. Participants were instructed to rest and avoid moderate or high levels of physical activity on days that they were not required to attend the laboratory.

### Test of maximal oxygen uptake

Maximal oxygen uptake was determined by an incremental test to exhaustion on a cycle ergometer (VeloTron and Velotron Coaching Software, Racermate, United States). Briefly, the test began with participants cycling at 80–90 rpm at 120 W, with the workload increasing by 20 W every min until volitional exhaustion or when cadence was unable to be maintained above 80 rpm. Expired gases were collected via a one-way breathing system (Hans-Rudulph, United States) and analyzed by a calibrated Moxus Metabolics Measurement cart (AEI Technologies, United States). Attainment of *V*O_2_ max was determined by the satisfaction of standard criteria (Midgley et al., 2007).

### Heat stress test

The heat stress test was of similar design to earlier work (Garrett et al., 2009; Lorenzo et al., 2010) and comprised cycling for 3 × 10 min submaximal workloads with a 3 min rest period between workloads, followed by a 5-km self-paced TT. Following a 5 min standardized warm-up, the participants completed three 10 min workloads at 50, 60, and 70% of their peak wattage corresponding to their individualized *V*O_2_ max. After the 70% workload was complete, a 5 min rest period was given before the start of the TT. Participants were able to view their rpm and were informed of the distance traveled every 500 m to assist with pacing. Heart rate (RS400, Polar Elektro, Finland), and core temperature (T_c_) (ttec 501-3 data logger and data logger software version 10.1, Nordex Pty Ltd, Australia; MEAS 449 1RJ rectal temperature thermistor, Measurement Specialities, United States) were sampled at 5 s intervals. Fluid intake (water, *ad libitum*), rating of perceived exertion (Borg RPE 6–20, Borg, 1970) and thermal comfort (TComf) were recorded throughout the test. Nude dry body mass was recorded pre and post-exercise on a calibrated set of scales (BF-522W, Tanita, Japan) and body mass was adjusted for fluid loss and expressed as a percentage change.

### Blood collection

Upon arrival at the laboratory, participants rested for 20 min before blood collection was performed. Blood was drawn in a seated position 10 min before and 10 min after each HST via a 22 g needle from a prominent superficial forearm vein located at the antecubital fossa, and drained directly into an 8.5 ml sterile serum separator Vacutainer tube containing a clot activator and gel for serum separation (Beckton and Dickson, USA). Samples were refrigerated at 4°C for 30 min to allow clotting and then centrifuged at 1000 × g at 6°C for 10 min (Rotina 420R, Hettich, Germany). Serum was removed and stored in 400 μl aliquots that were frozen immediately for a maximum of 3 months at −80°C for later analysis. Serum concentrations of IL-6 (Quantikine HS600B, R&D Systems, United States), IgM (AB137982, Abcam PLC, United Kingdom), and LPS (HIT302, Hycult, Biotechnology, Netherlands) were analyzed in duplicate by ELISA according to manufacturer's instructions.

### Aerobic interval training

Participants in HOT and NEUTRAL undertook matched aerobic interval training on a cycle ergometer (Monark Ergomedic 828 E, Sweden) in hot and humid (35°C and 70% RH) or thermo-neutral conditions (20°C and 50% RH), respectively. The exercise-training intervention included seven training sessions comprised a standardized 3 min warm-up followed by 4 × 10 min interval at a fixed workload of 55% *V*O_2_ max_._A 3 min rest period was given between each workload and water consumed *ad libitum*. A shorter duration interval-based protocol was chosen to better reflect the training status of the recreationally-trained participants; interval-based training has been shown to be beneficial for heat acclimation (Dawson et al., 1989; Kelly et al., 2016), and shorter duration training can reduce cumulative fatigue (Wingfield et al., 2016) while promoting performance (Nielsen et al., 1997). Heart rate was recorded at 5 s intervals and RPE and TComf recorded at the end of each interval. Participants self-reported symptoms of illness, inflection, soreness, or inflammation prior to the start of each training session. No symptoms of illness or infection were reported.

### Statistical analysis

Data that passed tests for homogeneity of variance were analyzed by a mixed-model analysis of variance or *t*-test (where appropriate) and significance accepted when *p* ≤ 0.05. Where significant differences were indicated they were identified with the *post hoc* Tukey Test. Data is expressed as mean ± SD and change scores expressed as mean ± 90% confidence limits (CL). The baseline TT performance (s) was not normally distributed and therefore analysis of covariance was used to investigate between-group differences with participant *V*O_2_ max employed as the covariate—TT results are expressed as adjusted mean ± SD or 90% CL where appropriate. Standardized effect sizes (ES) were calculated to indicate the magnitude of change and/or difference within- and between-groups. The criteria to interpret the magnitude of ES were: < 0.2 trivial, 0.2–0.6 small, 0.6–1.2 moderate, 1.2–2.0 large, and >2.0 very large (Hopkins, 2004).

Determination of biomarker concentrations and curve fit analysis was performed using GraphPad Prism Version 6.03 (GraphPad Software Inc, United States) according to the manufacturer's instructions. The manufacturer stated intra-assay precision was < 10% for all assays. Statistical analyses were performed in IBM SPSS Statistics Version 22 (IBM, United States). Power analysis was conducted prior to the study and a minimum of eight participants was deemed sufficient to detect the smallest worthwhile change between means assuming the reference change in 5 km TT performance was approximately twice the magnitude of the typical error of measurement, with a Type I error of 5 and Type II error of 20%.

## Results

### Heat stress test

#### Between group analyses

**Figure 2:**
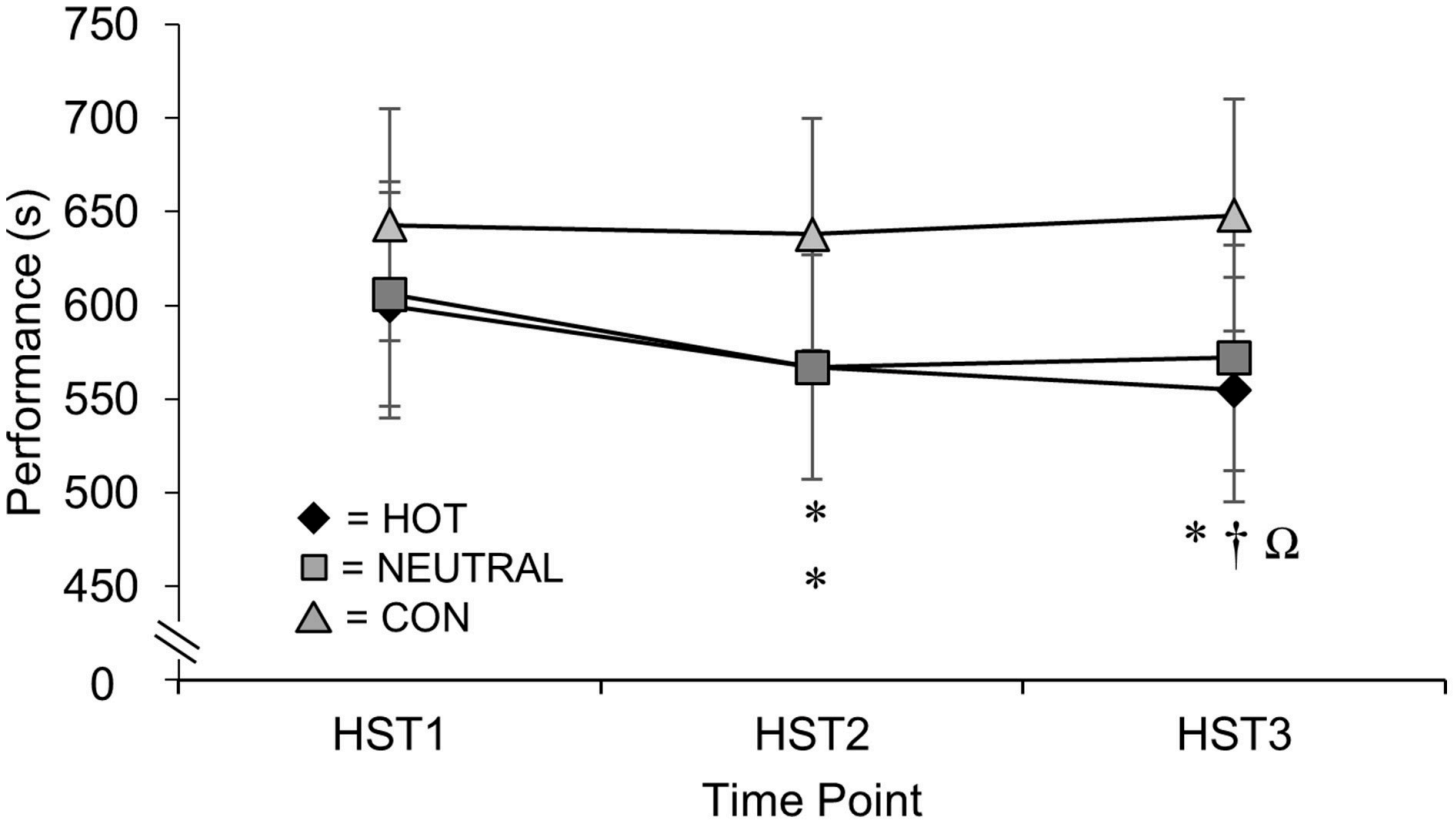
**Adjusted means ± SD of 5 km time trial performance (s) across heat stress tests (HST) 1, 2, and 3 for Heat (HOT), Thermo-neutral (NEUTRAL), and Control (CON) groups**. ^*^Faster from baseline. ^†^Faster than HST 2_._ ΩHOT was faster than CON.

**Figure 3:**
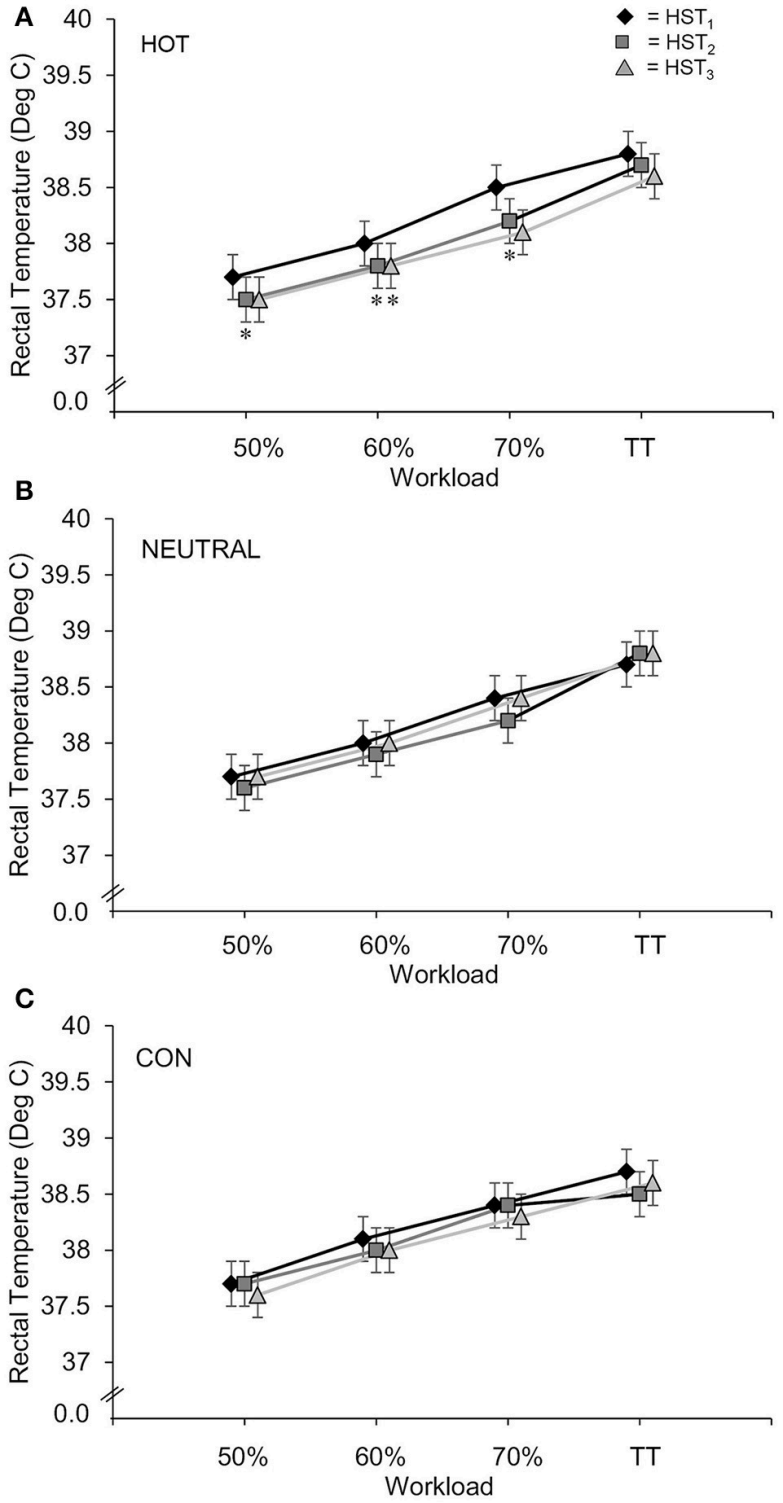
**Core temperature for Heat Training (HOT), Thermo-neutral Training (NEUTRAL), and Control (CON) groups during Heat Stress Tests (HST) 1, 2, and 3, expressed as mean ± SD**. ^*^Reduced from baseline at HST 2. †Reduced from baseline at HST 3.

At HST3 a significant between-group effect for TT was evident between HOT and CON (HOT was faster by 8.2%, ±5.2%, 90% CL, *p* = 0.03). Time trial performance is presented in Figure 2 as adjusted means from the analysis of covariance. No significant between-group effects of short-term heat training were observed for Tc (0.3 ± 0.6%, Figure 3), RPE, TComf, sweat loss, or HR (Table 1).

**Table 1:** **Physiological and perceptual responses to Heat Stress Tests**.

	**HST_1_**			**HST_2_**			**HST_3_**		
	**HOT**	**NEUTRAL**	**CON**	**HOT**	**NEUTRAL**	**CON**	**HOT**	**NEUTRAL**	**CON**
HR_50%_(bpm)	139 ± 15	135 ± 12	137 ± 14	136 ± 15	133 ± 11	138 ± 13	136 ± 17	133 ± 10	133 ± 13
HR_60%_(bpm)	162 ± 15	159 ± 9	157 ± 9	155 ± 14	154 ± 9	156 ± 9	155 ± 16	154 ± 11	153 ± 11
HR_70%_(bpm)	175 ± 13	178 ± 7	170 ± 8	169 ± 13	172 ± 9	170 ± 6	168 ± 13	171 ± 9	167 ± 7
HR _TT_(bpm)	177 ± 11	178 ± 9	169 ± 10	176 ± 9	179 ± 6	168 ± 7	179 ± 10	175 ± 10	164 ± 12
RPE_Avg_(units)	14 ± 1	14 ± 1	15 ± 1	13 ± 2	14 ± 2	13 ± 1	13 ± 2	15 ± 3	13 ± 2
RPE_End_(units)	17 ± 2	17 ± 2	17 ± 2	17 ± 2	18 ± 2	17 ± 3	17 ± 2	17 ± 2	16 ± 3
TComf_Avg_(units)	3.0 ± 0.5	3.0 ± 0.5	3.5 ± 0.5	2.0 ± 1.0^*^	3.0 ± 0.5	3.0 ± 1^Ω^	2.0 ± 1.0^*^^†^	3.0 ± 0.5^∞^	3.0 ± 0.5^*^^Ω^
TComf_End_(units)	4.0 ± 0.5	4.5 ± 0.5	4.5 ± 0.5	3.0 ± 1.0	4.5 ± 1.0^∞^	4.0 ± 1	3.0 ± 1.0^*^	4.0 ± 1.0	3.5 ± 1.0

*Data are expressed as mean ± SD. HOT, Heat training group; NEUTRAL, Thermo-neutral training group; CON, Control group; HR, Heart rate. Sweat loss (%) is expressed as the amount of sweat lost (kg) divided by the persons pre-exercise mass (kg) × 100. RPE_Avg_ and TComf_Avg_ are the mean Rating of Perceived Exertion and Thermal Comfort rating across the entire Heat Stress Test (HST). RPE_End_, and TComf_End_ represent the values recorded at the cessation of the HST*.

* *Significantly different from HST_1_*.

† *Significantly different from HST_2_*.

∞ *Significant difference between HOT and NEUTRAL*.

Ω *Significant difference between HOT and CON*.

#### Within group analyses

Both the HOT and NEUTRAL group significantly improved TT performance in HST_2_ at the end of the 7 days short-duration protocol (after four heat training sessions) compared to HST_1_, with HOT 33 ± 20 s (adjusted mean ± 90% CL) faster (*p* = 0.02) and NEUTRAL 39 ± 18 s faster (*p* = 0.01) than baseline. After conclusion of the post-training top-up period, only HOT had a significant improvement in their TT performance at HST_3_ compared to HST_1_, completing the course 45 ± 25 s faster (*p* = 0.01) compared to their HST_1_ performance. The performance of HOT in HST_3_ was also significantly improved from HST_2_ (12 ± 7 s, *p* = 0.01).

There was a small but significant mean reduction in exercising T_*c*_ observed in the HOT group from HST_1_ to HST_2_ during the 60% workload of −0.22 ± 0.14°C (mean ± 90% confidence limits, *p* = 0.02, ES = −0.53). Additionally, there was a trend for lower T_*c*_ during the 70% workload (−0.25 ± 0.21°C, *p* = 0.06, ES = −0.53) and during the TT (−0.25 ± 0.24°C, *p* = 0.09, ES = −0.45). Small-moderate significant reductions in T_*c*_ was observed in the HOT group from HST_1_ to HST_3_ at the 50%; −0.18 ± 0.10°C (*p* = 0.016), 60%; −0.23 ± 0.18°C (*p* = 0.04), and 70%; −0.34 ± 0.27°C (*p* = 0.05) workloads. The HOT group also experienced a small reduction in peak T_*c*_ during HST_2_ compared to HST_1_; −0.25 ± 0.21°C (*p* = 0.057), see Figure 3A. Neither the NEUTRAL nor the CON group experienced meaningful reductions in T_*c*_ in any of the HST's (Figures 3B,C).

The HOT group exhibited a moderate improvement in thermal comfort in HST_3_ compared to HST_1_ (*p* ≤ 0.01). Thermal comfort was also improved in HOT during HST_2_ and HST_3_ compared to NEUTRAL (*p* = 0.04 and *p* = 0.03, respectively). There were no meaningful within group reductions of HR during the HST's (Table 1).

### Inflammatory biomarker responses

#### Between-group analyses

No significant differences between groups in any of the biomarker responses were observed either at rest or in response to any of the three HST's. Between groups there was a ~8 ± 32% difference in post HST IL-6, ~52 ± 111% in LPS, and ~35 ± 36% in IgM.

#### Within-group analyses

**Figure 4:**
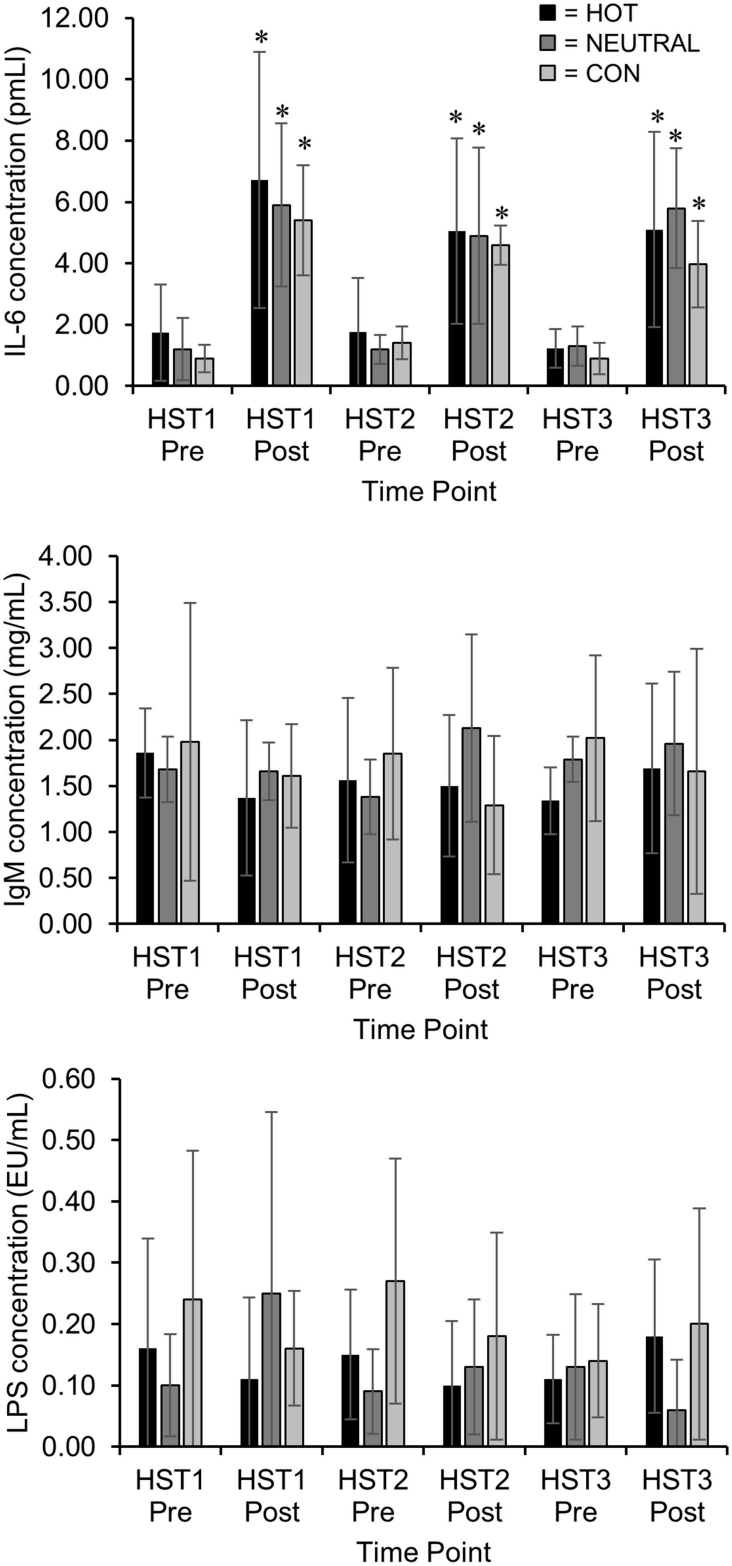
**Serum concentrations of interleukin 6 (IL-6), Immunoglobulin M (IgM), and Lipopolysaccharide pre and post Heat Stress Tests 1, 2, and 3**. ^*^ Increased from pre exercise concentration.

There was a large to very large (~4 ± 2 fold) rise in serum IL-6 concentration for all groups following each HST. Serum concentrations of IgM and LPS were not substantially different following the HST for each group and there were no significant time interactions observed in any group. However, there was a trend for a small reduction in post-exercise concentrations of IgM in all participants (*n* = 24) following the first HST (*p* = 0.08, ES = 0.40). There were no within-group changes observed in serum concentration of LPS (44 ± 208%) or IgM (6 ± 61%) neither pre nor post each HST. Blood biomarker concentrations are presented in Figure 4.

#### Training sessions

There were no within-group changes observed in exercising heart rate during each of the training sessions for the HOT or NEUTRAL groups. Although the HOT group exhibited higher HR in all training sessions compared to NEUTRAL. Table 2 outlines the physiological and perceptual variables collected during the interval training sessions.

**Table 2:** **Physiological and perceptual observations during sub-maximal aerobic interval training from training sessions one, four, and the third top up session**.

	**TR_1_**		**TR_4_**		**TU_3_**	
	**HOT**	**NEUTRAL**	**HOT**	**NEUTRAL**	**HOT**	**NEUTRAL**
HR (bpm)	161 ± 13	145 ± 9^∞^	157 ± 12	145 ± 6^∞^	154 ± 15	140 ± 13
RPE_Avg_ (units)	15 ± 1	15 ± 2	14 ± 2	15 ± 2	13 ± 3	13 ± 1^†^
TComf_Avg_ (units)	3.0 ± 1.0	3.0 ± 1.0	3.0 ± 1.0	3.0 ± 1.0	2.0 ± 1.0	3.0 ± 1.0

*Data is expressed as mean ± SD. HOT, Heat training group; NEUTRAL, Thermo-neutral training group; TR_1_, Training session on day one; TU_3_, Top up training session on day 15; HR, Mean heart rate across 4 × 10 min intervals. RPE_Avg_ and TComf_Avg_ are the mean Rating of Perceived Exertion and Thermal Comfort rating across the training session*.

† *Significantly different from TR_4_*.

∞ *Significant difference between HOT and NEUTRAL*.

## Discussion

Short term heat training followed by supplementary top-up sessions (seven training sessions over 18 days) improved TT cycling performance, reduced exercising core temperature, and improved thermal comfort during a strenuous cycling task in the heat. In contrast, participants in the thermo-neutral (exercise) and control conditions did not experience these physiological and perceptual improvements. However, as the thermo-neutral group also improved their 5 km TT performance after the initial short-term block of heat-training (5 training session in 7 days), it is likely a greater stimulus in terms of intensity and duration is required to elicit greater gains from heat training in shorter time periods. Although mean IL-6 concentration increased 4-fold following each HST, the exercise stimulus did not elevate other biomarkers of systemic inflammation such as IgM and LPS. As biomarker activity was largely unaffected by short-term heat training, as evidenced by IL-6 returning to basal level prior to each HST, it appears that it is possible to gain useful performance and thermoregulatory adaptations from short-duration training without compromising the immune system. Therefore, coaches and athletes can use short-term heat acclimation training coupled with supplementary heat training sessions to improve TT performance, in the confidence there is little likelihood of impairing immune system functionality.

Improvements in TT performance with short-term heat training have been reported by Lorenzo et al. (2010) in cycling and Garrett et al. (2012) in rowing. However, Garrett and colleagues did not include a control group undertaking matched training over the 5 day heat training program. It is possible that the improvement (−4 s) observed in 2000 m rowing time in that study could have been similar to that of an exercise alone control/placebo group. In our study the effects of heat training were largely similar to that of the exercise-alone group during the first week of training. However, the supplementary top-up sessions appeared to elicit further gains, indicating that while short term training offers some benefits a longer program offers additional benefits. In the study by Lorenzo and colleagues, one third of the experimental group (four out of twelve) were participants who had already completed the control condition of the experiment, consequently, the pre-exposure to exercise in the heat and heat stress test protocols. This prior exposure may have conferred a small degree of acclimation prior to taking part in the experimental portion of that study. In the present study, the inclusion of both an exercise matched (NEUTRAL) and control (CON) group allows clear interpretation of whether the heat acclimation training was responsible for the reported changes in performance and physiological adaptations. Adaptations and improvements reported previously (Lorenzo et al., 2010; Garrett et al., 2012); may relate to the increased frequency of training within a given training period. It is likely that the heat exposure resulted in ergogenic performance and thermoregulatory adaptations at the end of the 18 day period beyond that of exercise training alone.

The improved TT performance by participants in HOT was matched by those in NEUTRAL at HST_2_, indicating that the stimulus for performance gain over 7-days of short-duration training in moderately-trained individuals is exercise *per se* rather than the environmental conditions under which it is performed (i.e., hot or neutral). Although, there were additional performance gains for the HOT group after the three supplementary training sessions over 10 days which increased HOT's total heat load to nine exposures (two HST's and seven training sessions, approximately 9 h). Clearly, exercise in temperate conditions results in heat production which elevates body temperature (Gleeson, 1998), and among recreationally-active participants it seems probable that this heat production is a sufficient stimulus to generate modest adaptations over 7 days. The observation of continued adaptation and performance improvement only in the HOT group after the post-training top-up period (after the full 18 days) suggests that the generic adaptive responses experienced by NEUTRAL after 7 days had most likely run their course and plateaued. As this study recruited participants that were recreationally-active it is possible that elite endurance athletes, already well-accustomed to performing regular heat producing exercise would differentially experience greater gains compared to a matched neutral exercising group, although this remains to be determined.

Although a greater number of heat exposures (than imposed in this study) could yield more substantial physiological adaptations and performance improvements, it is also possible that this increase could trigger systemic inflammation (Lim et al., 2009). The ~4 fold increase of IL-6 concentration in all participants after the HST may not signify heat stress *per se*, but rather the stress invoked by the exercise demand itself. IL-6 can be released into the circulation following various pathological events such as physical exercise, trauma, sepsis, and thermal injury (Natelson et al., 1996; Moldoveanu et al., 2000). There are few studies that have investigated IL-6 as a blood biomarker during exhaustive exercise in the heat, although one study reported a very large increase in IL-6 following 2 h of exhaustive walking in protective clothing at 40°C (Selkirk et al., 2008). However, a different study reported a very large increase in IL-6 following 3 h of exercise at 60–65% of *V*O_2_ peak in typical laboratory conditions (Moldoveanu et al., 2000). Prolonged elevation of IL-6 may signify cumulative fatigue or a neuroinflammatory response (Vargas and Marino, 2014), however in the present study IL-6 returned to basal concentration prior to each HST. It appears the training load was adequate to elicit some physiological and performance benefits over the 18 day period, but not enough to elicit wider systemic or prolonged inflammation. Although IL-6 appeared to be the most sensitive blood biomarker to the exercise task, its usefulness in specifically signifying heat stress or acclimation status is limited given the non-heat specific nature of its response.

The low concentrations of LPS observed in this study indicates the participants tolerated the moderate-high heat load that was presented to them, and in doing so experienced minimal gut leakage (Pyne et al., 2014). As LPS is the primary endotoxin translocated to circulation under heat load (Yeh et al., 2013), its concentration and regulation is a primary consideration in study of responses to the heat. It appears that undertaking ~40 min of strenuous exercise in the heat is not sufficient to evoke a systemic inflammatory response in healthy moderately active individuals. Furthermore, as IgM is a key antibody in neutralizing LPS (Camus et al., 1998), its concentration in circulating blood can reflect the body's response to endotoxin accumulation and as protection against further challenges. In this study the pre- to post-exercise change in IgM concentration in the heat was not significant, however following the first HST there was a trend (*p* = 0.08) toward reduced concentrations in all participants. It is likely that a substantial heat and/or exercise stimulus may be required for IgM concentrations to be substantially affected, although in this case it seems possible that there was some degradation of the antibody occurring. Although some between changes were observed in LPS and IgM concentrations (44 and ~35% respectively) there was substantial uncertainty in these estimates due to high variability in the biomarker response. Only one other study has investigated the response of non-specific IgM following exercise in hot and humid conditions (Hailes et al., 2011). During that study a 20% increase of plasma IgM was reported pre- to post-exercise at day one of the heat acclimation program, this change was not present at day five, with post-exercise IgM not varying from basal levels (Hailes et al., 2011). The initial change of IgM in Hailes and colleagues' study may relate to the participants required to reach a terminal core temperature of 39.5°C, whereas in the present study core temperatures did not consistently rise to that level. Despite a substantial exercise and heat load (60 min HST), participants in the present study were able to cope with the demands of the exercise task with limited inflammation and immune disturbances.

## Conclusions

Short-term heat training with the addition of supplementary top-up training sessions over 18 days enhanced time-trial performance by ~9% in recreationally-active healthy adults, although thermo-neutral exercise training alone was a sufficient stimulus for performance gains of ~6% over 7 days. The effects of heat training appear to become more worthwhile between 7 and 18 days. Nevertheless, training in either the heat or neutral conditions proved beneficial to performance and thermoregulatory responses compared to a control (non-exercise) condition. However, none of the experimental groups exhibited substantial changes in LPS, IgM, or IL-6 indicating the training and heat load did not elicit an immune response. It is possible that a more intense heat training protocol may lead to greater physical and immune responses.

## Author contributions

JG, DP, GD, CM, and AE contributed to the study design. JG completed data collection and conceptualization and drafting of the article. JG and KM completed Biomarker analysis. All authors performed all data analysis and conceptualizing and revising the study critically for important intellectual content, and approved the final manuscript.

## Funding

This project was funded by an internal research grant from James Cook University.

### Conflict of interest statement

The handling Editor declared a past co-authorship with the authors JG and AE and states that the process nevertheless met the standards of a fair and objective review. The other authors declare that the research was conducted in the absence of any commercial or financial relationships that could be construed as a potential conflict of interest.
